# CNS-Draining Meningeal Lymphatic Vasculature: Roles, Conundrums and Future Challenges

**DOI:** 10.3389/fphar.2021.655052

**Published:** 2021-04-28

**Authors:** Sofia Pereira das Neves, Nickoleta Delivanoglou, Sandro Da Mesquita

**Affiliations:** Department of Neuroscience, Mayo Clinic, Jacksonville, FL, United States

**Keywords:** meninges, lymphatic vessels, central nervous system, drainage, cerebrospinal fluid, aging, disease, neuroinflammation

## Abstract

A genuine and functional lymphatic vascular system is found in the meninges that sheath the central nervous system (CNS). This unexpected (re)discovery led to a reevaluation of CNS fluid and solute drainage mechanisms, neuroimmune interactions and the involvement of meningeal lymphatics in the initiation and progression of neurological disorders. In this manuscript, we provide an overview of the development, morphology and unique functional features of meningeal lymphatics. An outline of the different factors that affect meningeal lymphatic function, such as growth factor signaling and aging, and their impact on the continuous drainage of brain-derived molecules and meningeal immune cells into the cervical lymph nodes is also provided. We also highlight the most recent discoveries about the roles of the CNS-draining lymphatic vasculature in different pathologies that have a strong neuroinflammatory component, including brain trauma, tumors, and aging-associated neurodegenerative diseases like Alzheimer’s and Parkinson’s. Lastly, we provide a critical appraisal of the conundrums, challenges and exciting questions involving the meningeal lymphatic system that ought to be investigated in years to come.

## Introduction: The Lymphatic System

The lymphatic vasculature is an important component of the circulatory system that was initially described back in the 17^th^ and 18^th^ centuries, by the pioneering works of Gasparo Aselli, Olaus Rudbeck, Thomas Bartholin and Paolo Mascagni (Natale et al., 2017; Sandrone et al., 2019). The lymphatic system mediates the drainage of interstitial fluid (ISF) and regulates immune cell trafficking and surveillance in the vast majority of mammalian tissues including the intestine, lungs, heart, liver, diaphragm, skin, eye and even in the meninges that enclose the central nervous system (CNS) (Escobedo and Oliver, 2016; Petrova and Koh, 2018; Oliver et al., 2020; Petrova and Koh, 2020). The lymphatic vessels (LVs) are composed by lymphatic endothelial cells (LECs) that primordially arise by transdifferentiation from the endothelium of the cardinal vein during embryonic development (Francois et al., 2008; Nicenboim et al., 2015; Stone and Stainier, 2019). Commitment to the LEC lineage is dictated by vascular endothelial growth factor C (VEGF-C) signaling through vascular endothelial growth factor receptor 3 (VEGFR3) (Oh et al., 1997; Mäkinen et al., 2001; Mandriota et al., 2001). This signaling pathway induces the downstream activation of the transcription factor SOX18 and expression of the genes encoding for prospero-related homeobox 1 (PROX1), lymphatic vessel endothelial hyaluronan receptor-1 (LYVE-1), podoplanin (PDPN) and C–C motif chemokine ligand (CCL) 21 (Gunn et al., 1998; Banerji et al., 1999; Breiteneder-Geleff et al., 1999; Wigle and Oliver, 1999; Francois et al., 2008). In turn, the transcription factor PROX1 acts synergistically with the nuclear factor chicken ovalbumin upstream promoter transcription factor II (COUP-TFII) to enhance the expression of LEC-associated genes, namely Flt4 (the gene encoding VEGFR3), and suppress the expression of genes mediating the commitment to the blood endothelial cell lineage (Lee et al., 2009; Sabine et al., 2012). Signaling pathways involving bone morphogenetic protein 2 (BMP2), RAF1/MEK/ERK or Phospholipase C-γ (PLC-γ) have also been implicated in LEC lineage commitment and modulation of LV formation and function (Mancini and Toker, 2009; Norrmén et al., 2009; Coso et al., 2012; Deng et al., 2013; Dunworth et al., 2014). In particular, PLC-γ regulates CLEC2-PDPN signaling, and PLC-γ2 deficiency leads to lymphatic malformation, aberrant uncoupling of the lymphatic and blood vasculature and backflow of venous blood into the lymphatic vasculature (Sebzda et al., 2006; Ichise et al., 2009; Ichise et al., 2016). During embryonic stages, differentiated LECs organize to form the so-called lymph sacs, which subsequently foster the sprouting and development of the lymphatic capillaries (Lee and Suami, 2020) that attach to the extracellular matrix, by interaction with anchoring filaments (Tammela and Alitalo, 2010). Further sprouting, pruning and cellular specialization refine the lymphatic system in a well-organized network that, together with the capillaries, consists of larger vessels (pre-collecting and collecting lymphatics containing specialized valves) and lymph nodes (LNs). Although most of the peripheral lymphatic vasculature forms during embryonic development, lymphatic sprouting and lymphangiogenesis can also take place after birth and in adulthood (Oliver et al., 2020; Petrova and Koh, 2020). Recent experimental evidence points to two main mechanisms of post-developmental lymphangiogenesis: proliferation of LECs (from pre-existing lymphatics) and cellular transdifferentiation. Bone marrow-derived cells, myeloid precursors, blood-forming hemogenic endothelial cells (from the dermal blood capillary bed) and heart-associated progenitor cells are amongst the cell types that can efficiently transdifferentiate into LECs (Mahadevan et al., 2014; Klotz et al., 2015; Martinez-Corral et al., 2015; Stanczuk et al., 2015; Pichol-Thievend et al., 2018; Maruyama et al., 2019; Lioux et al., 2020).

Lymphatic capillaries, also named initial LVs, are thin-walled and blind-ended vessels formed by a monolayer of LECs that may respond to higher levels of VEGF-C to initiate lymphangiogenesis (Pfeiffer et al., 2008; Lohela et al., 2009). Initial LVs have small button-like junctions, a discontinuous basement membrane and lack pericytes and smooth muscle cells (SMCs) (Dejana et al., 2009). The button-like junctions and anchoring filaments present in initial LVs constitute the primary lymphatic valves that enable the entry of macromolecules and immune cells (Dejana et al., 2009; Tammela and Alitalo, 2010). Initial LVs converge into pre-collecting and then collecting LVs, which are bigger in caliber and are surrounded by contractile SMCs. Collecting LVs are almost impermeable structures composed by tight and continuous zipper-like junctions and secondary intraluminal valves that prevent lymph backflow and promote unidirectional drainage (Petrova and Koh, 2020). LECs that form the collecting lymphatics express specific markers like FAT4, Ras interacting protein 1 (RASIP1) and CCL27, alongside other proteins that modulate secondary valve development and function, like Forkhead box protein C2 (FOXC2), Ephrin type-B 2, GATA-binding factor 2 (GATA2), PLC-γ and nuclear factor of activated T cells 1 (NFATc1) (Norrmén et al., 2009; Sabine et al., 2012; Brouillard et al., 2014; Kazenwadel et al., 2015; Liu et al., 2018; Betterman et al., 2020). The low-pressure unidirectional transportation of lymph fluid, cells and macromolecules is also facilitated by skeletal muscle contraction, arterial vasomotion, respiration (Bazigou and Makinen, 2013) and by mechanical forces like fluid pressure and shear stress (Tzima et al., 2005). Lymph drained from different peripheral organs circulates through the lymphatic vascular network and is ultimately drained back into the venous blood circulatory system via the thoracic duct (Oliver et al., 2020).

The lymphatic vasculature collects and transports various types of antigens and immune cells (such as T and antigen-presenting cells) from tissues into their respective draining LNs, thus contributing to immune surveillance and resolution of inflammation (Huggenberger et al., 2011; Onder et al., 2017; Oliver et al., 2020; Petrova and Koh, 2020). The initial and collecting LVs secrete CCL21 and CCL27, respectively, which bind to their distinct receptors C–C motif chemokine receptor (CCR) 7 and CCR10 expressed by different immune cell types, including activated dendritic cells (DCs), T and B cells. In response to CCL21 or CCL27 gradients, immune cells are recruited to the vicinity of LVs and initiate their migration into the LNs (Forster et al., 2008; Wick et al., 2008; Ulvmar et al., 2014). At the LNs, recruited and resident immune cell populations engage and orchestrate immune responses that may culminate in the activation, proliferation and migration of antigen-specific T and B cells into the blood circulation and homing into affected tissues. Alternatively, the process of lymphatic trafficking, egress and immune cell activation at the draining LNs can also be regulated by other proteins expressed by LECs, namely sphingosine-1-phosphate (S1P), colony-stimulating factor–1 (CSF1), vascular cell adhesion molecule 1 (VCAM-1) and intercellular adhesion molecule 1 (ICAM-1) (Pham et al., 2010; Huggenberger et al., 2011; Teijeira et al., 2017; Petrova and Koh, 2018; Mondor et al., 2019).

Severe lymphatic vasculature malfunction can seriously compromise the drainage of ISF and lead to the development of lymphedema. Lymphedema is a disabling and potentially life-threatening pathological condition characterized by tissue edema and swelling, exacerbated buildup of adipose tissue, compromised wound healing capacity and a dysfunctional immune system that often leads to recurrent infections (Rockson, 2001; Oliver et al., 2020). Primary lymphedema is hereditary and results from genetic anomalies that lead to deficient levels of proteins that regulate lymphangiogenesis and lymphatic function, including VEGFR3, FOXC2, SOX18, collagen and calcium binding EGF domains 1 (CCBE1), ADAM metallopeptidase with thrombospondin type 1 motif 3 (ADAMTS3) and GATA2 (Fang et al., 2000; Irrthum et al., 2000; Irrthum et al., 2003; Alders et al., 2009; Kazenwadel et al., 2012; Brouillard et al., 2017; Jones and Mansour, 2017; Oliver et al., 2020). Secondary lymphedema results from damage to the lymphatic system that can be caused either from infections, such as filariasis, or certain therapeutic interventions, like surgical resection of LNs or tumors (Rockson, 2001; Pfarr et al., 2009; Oliver et al., 2020).

In 2015, two seminal studies have shown that the lymphatic system reaches even the border meningeal tissues of the CNS (Aspelund et al., 2015; Louveau et al., 2015). This recent (re)discovery of meningeal lymphatics led to a better understanding about the drainage of brain and spinal cord fluids and solutes (Antila et al., 2017; Louveau et al., 2018; Jacob et al., 2019) and provided a new vascular system that might be easily harnessed to treat brain insults and combat neurodegenerative diseases such as Alzheimer’s and Parkinson’s. In the following sections of this manuscript, we will provide an overview of the structural and functional roles of meningeal lymphatics in health and disease and propose hypotheses to be tested in future studies addressing the meningeal lymphatic system.

## Development, Cytoarchitecture and Morphology of Meningeal Lymphatics

Despite the early, but inconclusive, reports about the existence of a lymphatic-like system involved in cerebrospinal fluid (CSF) drainage to peripheral LNs (Brierley and Field, 1948; Bradbury and Cole, 1980; Bradbury et al., 1981; Szentistvanyi et al., 1984; Erlich et al., 1986; Andres et al., 1987; Yamada et al., 1991; Kida et al., 1993; Boulton et al., 1996; Killer et al., 1999; Silver et al., 1999; Gausas et al., 2007), the cytoarchitecture and functional features of the lymphatic vasculature present in the CNS meninges of both rodents and humans have only recently been systematically characterized (Aspelund et al., 2015; Louveau et al., 2015; Absinta et al., 2017; Jung et al., 2017; Da Mesquita et al., 2018b; Visanji et al., 2018).

**FIGURE 1:**
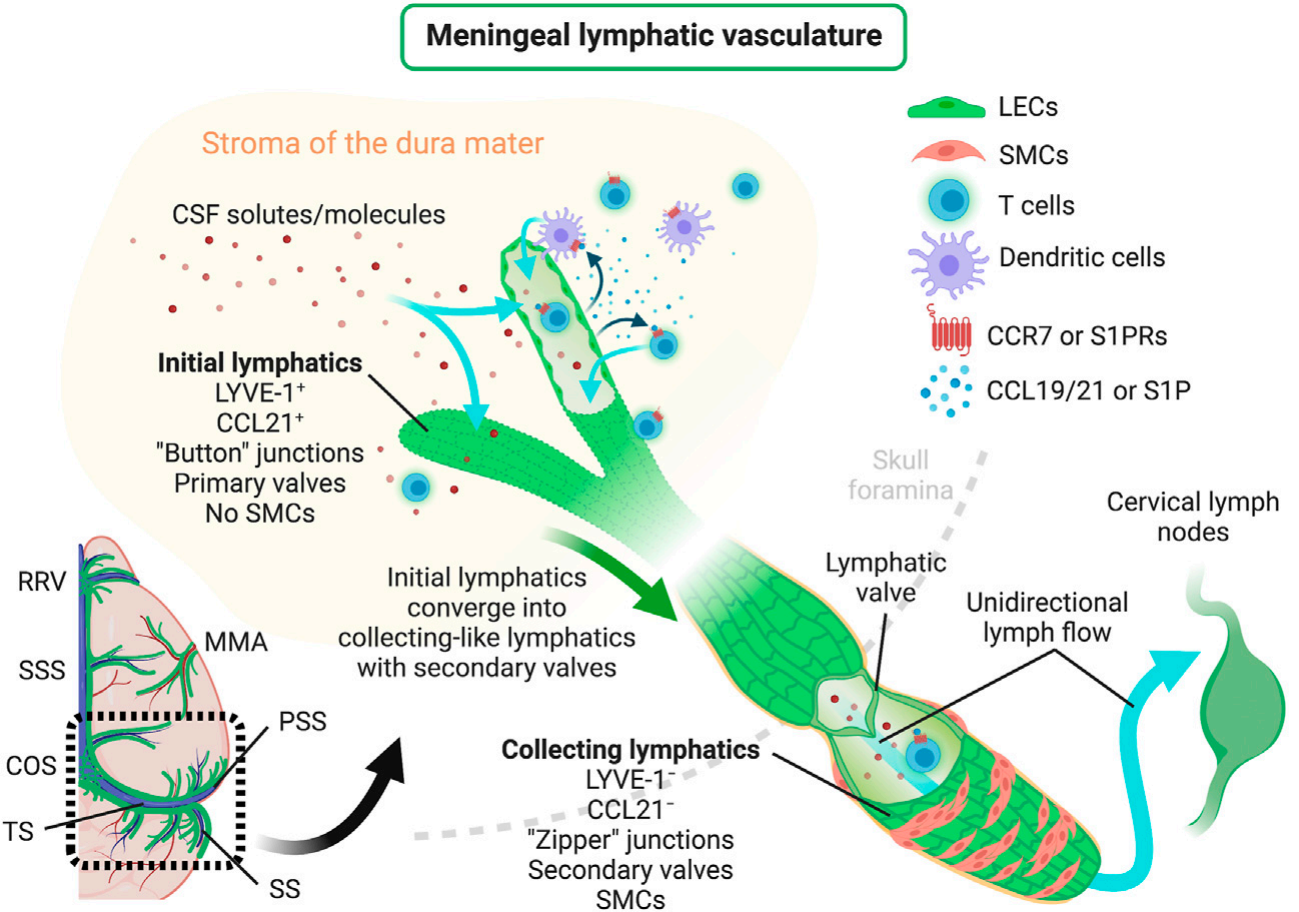
Scheme depicting the structure of meningeal lymphatic vessels, lymphatic drainage of CSF solutes/molecules, mechanisms regulating the trafficking of immune cells through meningeal lymphatics and the unidirectional lymphatic flow into the brain draining CLNs. CCL19/21, C–C motif chemokine ligand 19/21; CCR7, C–C motif chemokine receptor 7; COS, confluence of sinuses; CSF, cerebrospinal fluid; LECs, lymphatic endothelial cells; LYVE-1, lymphatic vessel endothelial hyaluronan receptor-1; MMA, middle meningeal artery; PSS, petrosquamosal sinus; RRV, rostral rhinal vein; S1P, sphingosine-1-phosphate; S1PRs, sphingosine-1-phosphate receptors; SMCs, smooth muscle cells; SS, sigmoid sinus; SSS, superior sagittal sinus; TS, transverse sinus. Created with BioRender.com.

In mice, meningeal LVs develop postnatally and become fully mature around postnatal day 28 (Antila et al., 2017), contrarily to most peripheral lymphatic vasculature that develops during embryonic stages, beginning at embryonic days 9–10 [peripheral lymphatic development was recently reviewed in detail by others (Gutierrez-Miranda and Yaniv, 2020)]. The experimental data available so far suggests that meningeal LECs originate from venous precursor cells, which are somewhat distinct from the ones that give rise to skin and intestinal lymphatics (Martinez-Corral et al., 2015; Stanczuk et al., 2015; Antila et al., 2017), in a process that is VEGFR3-dependent (Aspelund et al., 2015). In fact, loss-of-function studies have shown that both the development and maintenance of adult meningeal LVs are dependent on VEGF-C signaling through VEGFR3, but not on VEGF-D signaling (Antila et al., 2017). However, a more recent study has shown that mice lacking PLC-γ2 also present a structurally and functionally impaired meningeal lymphatic system, thus suggesting that meningeal lymphatic development might not be exclusively dependent on VEGFR3 signaling (Bálint et al., 2020). Primordial meningeal LECs stem from and around the veins at the skull foramina and later extend from the basal into the lateral and dorsal meninges along the major arteries (pterygopalatine and middle meningeal arteries) and venous sinuses (Aspelund et al., 2015; Antila et al., 2017; Izen et al., 2018; Ahn et al., 2019). In adult mice, the meningeal initial LVs are found lining the rostral rhinal vein, superior sagittal sinus, confluence of sinuses, transverse sinus, pterygopalatine artery and middle meningeal artery (Figure 1) (Aspelund et al., 2015; Louveau et al., 2015; Louveau et al., 2018; Ahn et al., 2019). The LECs that form the meningeal initial LVs also express the specific markers PROX1, LYVE-1, VEGFR3, PDPN and CCL21, but lack integrin α-9 which is involved in valve formation and is characteristically expressed in the meningeal collecting LVs (Antila et al., 2017; Louveau et al., 2018; Ahn et al., 2019). Initial LVs from the dorsal meninges converge to larger collecting-like lymphatics at the basal dural meninges that have tighter zipper-like junctional patterns, integrin α-9-expressing valves, but lack SMCs, thus resulting in a lymphatic network that extends throughout the brain meningeal dura layer (Aspelund et al., 2015; Louveau et al., 2018; Ahn et al., 2019). The collecting-like LVs are mostly found in the meninges attached to the lateral and basal parts of the skull, lining the petrosquamosal and sigmoid sinuses. Meningeal collecting-like LVs extend along the jugular vein and exit the skull through the foramina and merge into peripheral collecting LVs, which are wrapped by SMCs and drain primarily to the deep cervical LNs (CLNs) and later to the superficial CLNs (Figure 1) (Aspelund et al., 2015; Antila et al., 2017; Louveau et al., 2018; Ahn et al., 2019).

The vertebral meningeal LVs that wrap the spinal cord also develop postnatally, through a VEGF-C–VEGFR3-dependent stemming process, which starts at the spinal canal and gives rise to mature LVs at the vertebrae and intervertebral spaces from the cervical, thoracic and lumbar regions (Antila et al., 2017; Louveau et al., 2018; Jacob et al., 2019). Despite presenting distinct organizations and morphologies, the brain and spinal cord meningeal LVs are connected around the foramen magnum and at the cisterna magna (Antila et al., 2017; Jacob et al., 2019). Careful anatomical examinations involving Prox1 reporter mice and staining for different LEC-specific markers has shown that the architectural organization of the vertebral meningeal LVs is segmented, meaning that each vertebral disc is sheathed by two dorsal and two ventral semicircular lymphatic branches that are interconnected by thinner and elongated LVs (Jacob et al., 2019). Moreover, the vertebral meningeal LVs are in direct contact with nerves and ganglia, exit at the intervertebral foramina to connect to peripheral lymphatics and can drain into cervical, thoracic and lumbar LNs (Villaro et al., 1987; Miura et al., 1998; Holzmann et al., 2015; Ma et al., 2019a; Jacob et al., 2019).

Unlike the primordial non-lumenized LV-like structures (formed by mural LECs) found in the zebrafish meninges (Bower et al., 2017), the meningeal lymphatic vasculature found in rodents, non-human primates and humans shows quite similar marker expression, morphologies and topographies (Louveau et al., 2015; Absinta et al., 2017; Visanji et al., 2018). Despite recent reports showing VEGFR3-expressing LECs imbedded in the leptomeninges (Shibata-Germanos et al., 2020), and lymphatic processes reaching the pia mater (Cai et al., 2019), the current understanding is that the mature meningeal LVs are anatomically confined to the dura mater layer under homeostatic conditions (Louveau et al., 2015; Antila et al., 2017; Louveau et al., 2018; Jacob et al., 2019). However, this is still controversial and more in-depth studies are required to fully understand the anatomy of the mammalian meningeal lymphatic vasculature.

Noticeably, aging affects meningeal lymphatic morphology and function (Da Mesquita et al., 2018b), but spares the lymphatics found in the diaphragm, skin, ear and trachea (Ahn et al., 2019). In the next sections we will focus more on the aging-associated changes in the meningeal lymphatic system and their impact in diseases that affect the CNS.

## Meningeal Lymphatic Drainage and Brain Physiology in Adulthood and Aging

The existence of a *bona fide* meningeal lymphatic system led to a reassessment of CNS drainage routes and mechanisms. Outflow of fluids (and solutes) from the mammalian CNS is thought to occur via 3 main pathways: 1) the arachnoid granulations and villi at the venous sinuses in the meningeal dura mater (Weller, 1998), 2) the peripheral lymphatics in the vicinity of the cribriform plate (Bradbury and Westrop, 1983; Furukawa et al., 2008) and 3) the meningeal lymphatic vasculature (Aspelund et al., 2015; Louveau et al., 2015). Nevertheless, recent static and dynamic imaging studies strongly suggest that the major route of CSF outflow is via meningeal LVs rather than the nasal lymphatics or dural venous sinuses (Ma et al., 2017; Ahn et al., 2019; Kutomi and Takeda, 2020; Melin et al., 2020). In fact, the process of CSF drainage into the deep and superficial CLNs is dependent on the integrity of the meningeal lymphatic vascular network and was reduced: 1) upon photodynamic ablation of meningeal lymphatics, 2) in Prox1 heterozygous mice, which present mispatterned and leaky LVs (Harvey et al., 2005), and 3) upon surgical ligation of afferent lymphatics of the deep CLNs (Da Mesquita et al., 2018b; Louveau et al., 2018). However, reduction of CSF outflow into the deep CLNs did not significantly affect the drainage into the superficial CLNs, suggesting that alternative routes of lymphatic drainage might exist and should be explored in future studies (Louveau et al., 2018). The involvement of the dorsal meningeal initial lymphatics in CSF outflow is also somewhat debatable, mostly due to their small diameter, simple morphology and lack of intraluminal valves (Ahn et al., 2019). However, the dorsal initial lymphatics and the larger collecting-like basal lymphatics are part of the same meningeal lymphatic network—one where the initial lymphatics converge into collecting-like and then collecting LVs that exit the skull and are connected to the deep CLNs (Aspelund et al., 2015). In fact, several independent studies support the essential role of both the dorsal initial and basal collecting-like meningeal lymphatics in the drainage of brain-associated fluids, solutes and cells (Da Mesquita et al., 2018b; Louveau et al., 2018; Ahn et al., 2019; Liu X. et al., 2020; Hu et al., 2020; Song et al., 2020).

The meningeal lymphatic vasculature does not contact directly with the brain parenchyma under physiological conditions (Da Mesquita et al., 2018a). In the brain, fluid circulation, exchange and recycling are mediated by the glymphatic system, which consists of a perivascular route delimitated by the astrocytic end feet (Iliff et al., 2012; Jessen et al., 2015). This brain perivascular pathway was initially described by Rennels and colleagues (Rennels et al., 1985), but only fully characterized and termed “glymphatic system” latter on via state-of-the-art imaging and functional studies (Iliff et al., 2012; Iliff et al., 2013a; Xie et al., 2013). Besides being involved in the clearance of solutes and metabolites from brain parenchyma back into the subarachnoid spaces, the glymphatic system also participates in the regulation of water, ion and cholesterol homeostasis (Jessen et al., 2015). Moreover, glymphatic function is known to be dependent on aquaporin 4 (AQP4) expression at the astrocytic endfeet (Iliff et al., 2012), as well as on body posture and respiratory and vasomotor pulsations (Klose et al., 2000; Iliff et al., 2013b; Lee et al., 2015). Molecular clearance through the glymphatic pathway in mice is increased during sleep (or under anesthesia) and decreased when the animals are awake (Xie et al., 2013; Hablitz et al., 2020). This feature is attributed, at least in part, to increased ISF volume (Berridge and Waterhouse, 2003) and increased AQP4 polarization to vascular astrocytic endfeet during sleep states (Hablitz et al., 2020).

Interestingly, the influx and efflux of CSF macromolecules through the glymphatic system was significantly impaired in adult mice with dysfunctional meningeal lymphatics as a result of photodynamic ablation of meningeal lymphatics, surgical ligation of the lymphatics afferent to the deep CLNs or Prox1 haplodeficiency (Da Mesquita et al., 2018b). These observations led to the conclusion that, although physically detached, the glymphatic and meningeal lymphatic systems are functionally connected (Da Mesquita et al., 2018a; Da Mesquita et al., 2018b). However, contrarily to glymphatic influx/efflux, meningeal lymphatic drainage of CSF content seems to be increased when mice are awake (Ma et al., 2019b; Hablitz et al., 2020), through a mechanism that is under circadian control, but does not dependend on the light/dark cycle (Hablitz et al., 2020). However, it is still unclear whether changes in the sleeping pattern impact on meningeal lymphatic function.

Meningeal lymphatics modulate the drainage of immune cells from the CNS meninges (Louveau et al., 2018; Hsu et al., 2019). Recent studies indicate that this process is mediated, at least in part, by CCR7 signaling (Figure 1), since the migration of T cells and DCs from the meningeal compartment into the deep CLNs was significantly reduced in Ccr7-null mice compared to wild type controls (Louveau et al., 2018). Yet, the chemoattractant S1P has also been implicated in immune cell trafficking through lymphatics (Czeloth et al., 2005; Rathinasamy et al., 2010) and it is still unclear whether the beneficial effects of S1P receptor modulators in the context of CNS autoimmunity, like fingolimod, [reviewed elsewhere (Chun and Hartung, 2010)] are achieved by blocking lymphatic drainage of leukocytes at the CNS meninges. Likewise, it is well established that the activation of peripheral LVs by proinflammatory cytokines, such as tumor necrosis factor (TNF), can induce the upregulation of leukocyte adhesion molecules, such as ICAM-1, VCAM-1 and E-selectin, that affect immune cell drainage and egress into LNs (Johnson et al., 2006). However, there is little evidence about the consequences of adhesion molecule overexpression in the activated meningeal lymphatic vasculature. In sum, the described evidence underlines the close involvement of the meningeal lymphatic system in the modulation of immune cell trafficking and the mechanisms that, if therapeutically targeted, might influence CNS immune cell drainage and activation.

Developing imaging tools has proven to be critical for the better understanding of meningeal lymphatic function in the human CNS. Different magnetic resonance imaging (MRI) protocols have been recently established to characterize the anatomy and function of meningeal LVs in humans. Some of these imaging techniques seem to allow the simultaneous measurement of CSF drainage into the CLNs, as well as the circulation of CSF/ISF solutes through the glymphatic pathway, under physiological and pathological conditions (Absinta et al., 2017; Ringstad et al., 2017; Taoka et al., 2017; Eide et al., 2018; Zhou W. et al., 2020; Zhou Y. et al., 2020; Ringstad and Eide, 2020; Yang et al., 2020; Ding et al., 2021). Alongside MRI, optical coherence tomography (OCT) has also been used to image meningeal LVs in animal models (Semyachkina-Glushkovskaya et al., 2017). However, this OCT protocol involved a transient step of blood-brain barrier (BBB) disruption, which means that, although promissing for longitudinal monitoring of lymphatic function (Gong et al., 2016), this technique would have to be revised in order to make it appropriate for the visualization of meningeal lymphatics in humans. The development or perfection of imaging techniques is critical to draw a comprehensive map of the meningeal lymphatic network in different mammalian species, and its contribution for waste clearance and immune cell trafficking in the CNS (Raper et al., 2016; Da Mesquita et al., 2018a).

Aging leads to a progressive impairment in glymphatic function that is accompanied by a decrease in dorsal meningeal LV diameter and coverage and, consequently, in reduced CSF outflow and macromolecule drainage into the deep CLNs (Kress et al., 2014; Ma et al., 2017; Da Mesquita et al., 2018b). The meningeal collecting-like LVs of older animals presented a lymphedematous phenotype characterized by increased size, higher number of branches, a dysmorphic distribution of type IV collagen and fewer lymphatic valves (Ahn et al., 2019). Interestingly, a similar decreased function of both the meningeal lymphatic and glymphatic systems was recently described in aged humans (Zhou Y. et al., 2020). Altogether, these findings suggest that a significant reduction of CNS lymphatic vascular drainage with aging is accompanied by deficits in CSF production (May et al., 1990; Liu G. et al., 2020) and fluid re-circulation through glymphatics (Kress et al., 2014; Zhou Y. et al., 2020). Of notice, boosting meningeal lymphatic function by inducing VEGF-C overexpression enhanced the cognitive performance of aged mice (Da Mesquita et al., 2018b). The beneficial effect of VEGF-C was abrogated in old mice that were also subjected to surgical ligation of the CSF-draining lymphatics afferent to the deep CLNs, which clearly showed that enhanced meningeal lymphatic drainage was responsible for the observed improvement in cognitive behavior (Da Mesquita et al., 2018b).

As we have described so far, the meningeal lymphatic vasculature is continuously draining the CNS, interacts with other mechanisms of brain cleansing and is severely affected by aging. We will next outline important experimental observations that support the close involvement of the meningeal lymphatic system in different neuropathological conditions that are closely linked with accumulation of toxic metabolic waste in the brain and persistent neuroinflammation. Additionally, we will discuss the possibilities of modulating meningeal lymphatic drainage to treat neurological disorders that preferentially afflict the elderly population.

## Role of Meningeal Lymphatic (dys)function in Neurological Disorders

**FIGURE 2:**
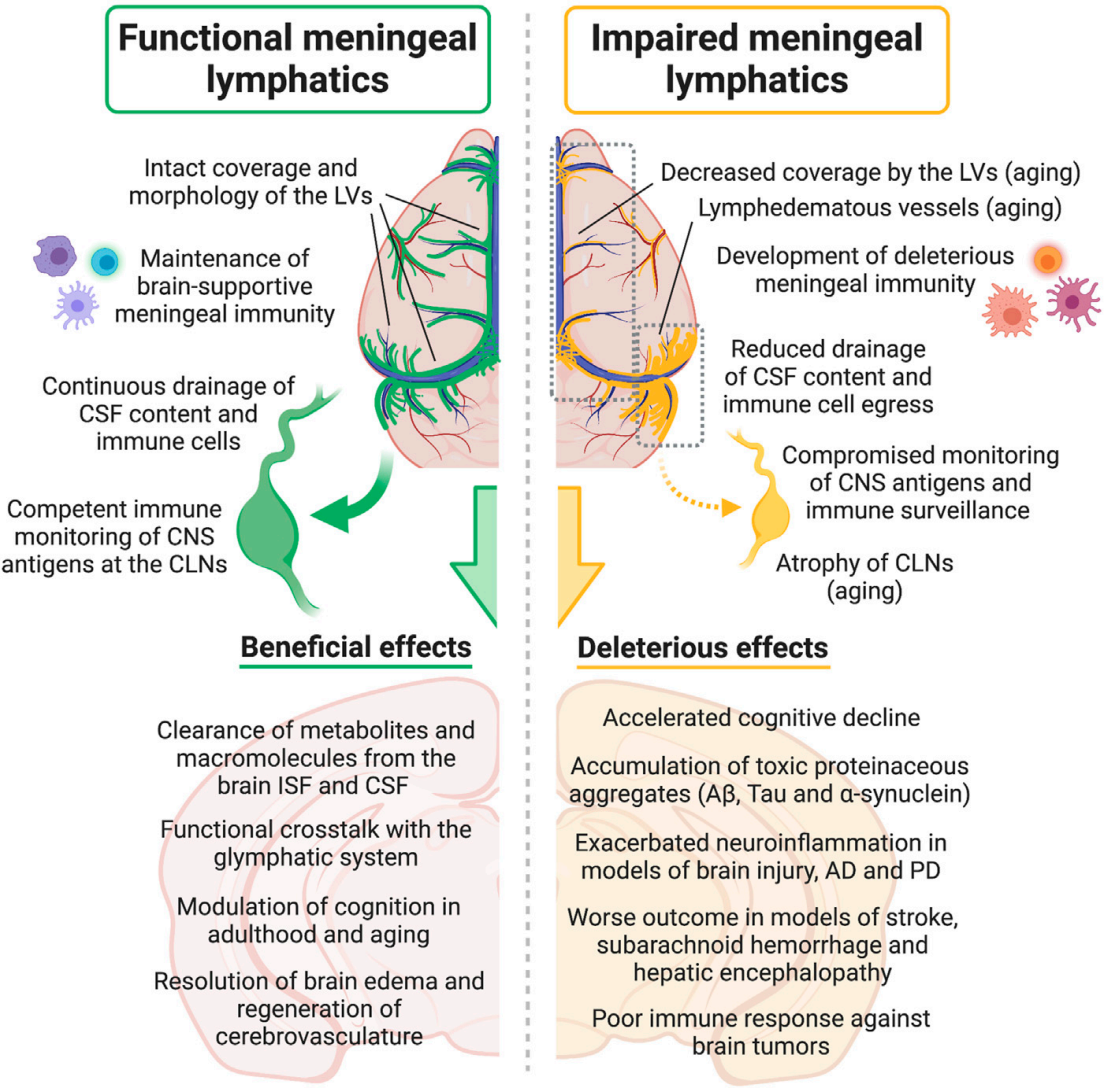
Effects of meningeal lymphatic (dys)function on CSF drainage, immune cell trafficking into the cervical lymph nodes, meningeal immunity, CNS immune surveillance, neuroinflammation and other neural processes, either under physiological conditions or in disease models. Aβ, amyloid beta; AD, Alzheimer’s disease; CNS, central nervous system; CSF, cerebrospinal fluid; CLNs, cervical lymph nodes; ISF, interstitial fluid; LVs, lymphatic vessels; PD, Parkinson’s disease. Created with BioRender.com.

Aside from its role in modulating CNS drainage and homeostasis, the meningeal lymphatic vasculature is intrinsically involved in the pathophysiology of different neurodegenerative diseases. As we will describe in the following sections, recent studies have shown that dampened cleansing of molecules prone to aggregation and reduced immune cell egress into the draining CLNs, as a result of dysfunctional CNS lymphatic drainage, can have a significant impact on disease outcome (Figure 2).

### Alzheimer’s Disease

The accumulation of extracellular amyloid plaques rich in aggregated amyloid beta (Aβ) peptides is a pathological hallmark of Alzheimer’s disease (AD) (Ittner and Gotz, 2011). Impairments in Aβ clearance mechanisms contribute to its accumulation, aggregation into toxic oligomeric intermediates and seeding into larger amyloid plaques (Wisniewski and Goni, 2014). Toxic Aβ species can be removed from the brain interstitium by several mechanisms, including degradation by enzymes (Qiu et al., 1998; Farris et al., 2003; Hernandez-Guillamon et al., 2015), phagocytosis by microglia and astrocytes (Weldon et al., 1998; Jones et al., 2013), receptor-mediated excretion across the BBB (Shibata et al., 2000; Silverberg et al., 2010), and perivascular efflux into CSF-filled compartments (namely the subarachnoid space) via the glymphatic system (Iliff et al., 2012). Importantly, Aβ accumulation can induce synaptic deficits (Terry et al., 1991; Mucke et al., 2000; Tsai et al., 2004), alterations in neuronal activity (Palop et al., 2007; Frazzini et al., 2016), neuronal network dysfunction (Kowalski et al., 2001; Palop et al., 2007; Ben-Nejma et al., 2019), and behavioral deficits, such as cognitive impairment (Henderson et al., 1989; Grady et al., 2008).

Increased levels of Aβ peptides in the CSF are detected early-on in AD patients and precede the increased Aβ plaque deposition in the brain parenchyma (Bateman et al., 2012). Interestingly, studies involving postmortem tissue from AD patients and AD transgenic mice have shown that increased levels of Aβ can be detected in the meningeal layers (Joachim et al., 1988; Da Mesquita et al., 2018b), namely in the dura mater adjacent to the superior sagittal sinus (Da Mesquita et al., 2018b), and in the CLNs (Pappolla et al., 2014). Altogether these observations show that Aβ peptides have access to anatomical locations harboring the meningeal LVs, which can then promote their drainage into the periphery (Da Mesquita et al., 2018b). This prompted for the assessment of meningeal lymphatic function in AD transgenic mouse models that recapitulate cerebral amyloidosis. Although meningeal lymphatic function was found to be intact at younger ages in both J20 and 5xFAD transgenic mice (Da Mesquita et al., 2018b), altered lymphatic drainage was observed at older ages in 5xFAD (Kwon et al., 2019) and APP/PS1 mice (Wang et al., 2019). These discrepant observations might be explained by the combined effects of aging and Aβ toxicity on the meningeal lymphatic system, a subject that warrants further investigation (Da Mesquita et al., 2018b; Kwon et al., 2019). Induction of early meningeal lymphatic dysfunction by photodynamic ablation in adult J20 and 5xFAD mice resulted in exacerbated Aβ pathology in the meninges and brain (Da Mesquita et al., 2018b). Likewise, reduction of meningeal lymphatic output by ligation of the LVs afferent to the deep CLNs had detrimental effects in the levels of forebrain soluble Aβ_1-40_ and Aβ_1-42_, Aβ deposition and gliosis in APP/PS1 mice (Wang et al., 2019). Importantly, reducing meningeal lymphatic ouflow led to spatial learning deficits in adult wild type mice (Da Mesquita et al., 2018b) and worsened short-term working memory in APP/PS1 mice (Wang et al., 2019).

Recent studies have also tested the effects of boosting the function of the meningeal lymphatics in mouse models of AD. Intracisternal injection of recombinant human VEGF-C promoted dural lymphangiogenesis in 9-month-old APP/PS1 mice, reducing the levels of soluble Aβ_1–40_ and Aβ_1–42_ in the brain and CSF, and improving cognitive performance (Wen et al., 2018). In a different study, delivery of a viral vector expressing mouse VEGF-C into the cisterna magna of J20 animals at 6–7 months of age did not improve the Aβ levels in the CSF, amyloid deposition in the hippocampus, or cognitive performance (Da Mesquita et al., 2018b). These discordant results might be explained by the different degrees of meningeal lymphatic vessel function or by the distinct kinetics of brain Aβ production and plaque development in APP/PS1 and J20 mice (Mucke et al., 2000; Jankowsky et al., 2004). The different methods and regimens of VEGF-C delivery employed in each study (Da Mesquita et al., 2018b; Wen et al., 2018) might also explain the different outcomes. More experimental evidence is needed to better understand if and how VEGF-C treatment can be beneficial to treat AD-associated brain pathology and symptoms.

Besides the extracellular deposition of Aβ plaques, another AD pathological hallmark is the presence of intracellular neurofibrillary tangles, which are composed by hyperphosphorylated forms of the microtubule-associated protein Tau (Ittner and Gotz, 2011). The exact mechanisms that lead to the formation and propagation of neurofibrillary tangles in AD remain elusive. A recent study has shown that Tau clearance from the CNS was impaired in the K14-VEGFR3-Ig genetic mouse model (Patel et al., 2019), which lacks a functional meningeal lymphatic vasculature due to impaired VEGF-C/D–VEGFR3 signaling (Aspelund et al., 2015; Patel et al., 2019). The authors showed that injection of fluorescently conjugated recombinant monomeric Tau into the hippocampus of K14-VEGFR3-Ig mice resulted in increased Tau retention in the brain at 48- and 72-h post-injection, when compared to control mice with intact meningeal lymphatics (Patel et al., 2019). In accordance, K14-VEGFR3-Ig mice also showed a delayed appearance of Tau in the blood (Patel et al., 2019), again highlighting the important role of the meningeal lymphatic system in brain-derived antigen outflow.

Altogether, there is a growing body of evidence supporting the notion that an impaired meningeal lymphatic drainage in AD could promote both Aβ and Tau accumulation in the brain, affecting disease severity and aggravating cognitive decline.

### Parkinson’s Disease

The accumulation of toxic forms of α-synuclein in Parkinson’s disease (PD) is closely linked with secondary pathological cascades that underlie the onset of clinical symptoms. One of the models used to study familial PD is the A53T transgenic mice that express the mutated A53T form of human α-synuclein (Giasson et al., 2002). Intracellular and extracellular aggregation of α-synuclein in the substantia nigra was significantly exacerbated in A53T mice after surgical ligation of the lymphatics draining into the deep CLNs (Zou et al., 2019). Increased α-synuclein aggregation after lymphatic ligation was accompanied by higher astrocytic activation, increased levels of proinflammatory cytokines, prominent loss of dopaminergic neurons and increased motor impairment, suggesting that decreased meningeal lymphatic drainage might affect different pathological features in the context of PD (Zou et al., 2019). Interestingly, A53T animals with ligated lymphatics also presented a decreased clearance of other macromolecules from the brain, namely of different toxic Tau species (Zou et al., 2019). Impairing the drainage of brain fluids and molecules into the deep CLNs in a PD model of α-synuclein fibril’s toxicity (induced by injecting α-synuclein fibrils into the brain) resulted in a higher accumulation and propagation of phosphorylated α-synuclein in the brain (Ding et al., 2021). Meningeal LVs have been recently implicated in the pathophysiology of human idiopathic PD. Using an MRI technique to measure meningeal lymphatic parameters, the authors were able to show that patients with idiopathic PD exhibited significantly reduced flow through the meningeal LVs along the superior sagittal sinus and sigmoid sinus, as well as a notable delay in the perfusion of deep CLNs by CSF-derived tracers, compared to atypical Parkinsonian patients. In fact, it was possible to accurately differentiate idiopathic PD and atypical Parkinsonian patients, with acceptable sensitivity and specificity, just by their profile of meningeal lymphatic outflow (Ding et al., 2021). Even though idiopathic PD and multiple system atrophy (one type of atypical Parkinsonian disorder) are both α-synucleinopathies, they are characterized by different species of misfolded α-synuclein (Shahnawaz et al., 2020), which highlights the need to understand whether the presence of distinct pathological α-synuclein aggregates in the CNS can have different effects on the meningeal lymphatic system. It will also be essential to explore if the modulation of meningeal lymphatic function (decreasing or increasing its drainage capacity) affects the neuroimmune response and behavior performance in PD (Joers et al., 2017; Butkovich et al., 2020). Additional experiments are also needed in order to understand if enhancing meningeal lymphatic drainage in PD might prove to be efficacious in alleviating not only brain α-synuclein pathology and neuroinflammation but also PD-related clinical symptoms, including motor deficits, sensory abnormalities, sleep disturbances, autonomic dysfunction, impaired gut motility, dysbiosis and fatigue (Pfeiffer, 2016; Chapelet et al., 2019; Prigent et al., 2019; Butkovich et al., 2020; Aho et al., 2021).

### CNS Autoimmune Disease

Multiple sclerosis (MS) is the most common autoimmune demyelinating disease of the CNS, whose main pathological mechanisms and therapies have been discovered with the aid of the experimental autoimmune encephalomyelitis (EAE) mouse model (Steinman and Zamvil, 2005). Induction of chronic EAE in mice did not result in significant alterations in meningeal lymphatic morphology or CSF drainage into the deep CLNs at an early stage of the disease (Louveau et al., 2018). However, peripheral lymphangiogenesis was observed in the cribriform plate at a later stage of chronic EAE (Louveau et al., 2018; Hsu et al., 2019), which suggested that a pathological peripheral lymphatic remodeling could be affecting disease severity in this model (Louveau et al., 2018; Hsu et al., 2019). Analysis of the brain meninges of EAE mice revealed a higher number of T cells inside and around the meningeal LVs after the appearance of clinical symptoms, when compared to healthy controls (Louveau et al., 2018). Accordingly, a decreased number of T cells in the deep CLNs was observed in a spontaneous EAE model, immediately before the appearance of clinical manifestations, suggesting that activation of antigen-specific T cells in these draining LNs might precede CNS T cell infiltration (Furtado et al., 2008). These observations prompted for a better assessment of the roles of the peripheral nasal lymphatics and the meningeal lymphatics in the initiation and progression of disease in the EAE model (Louveau et al., 2016; Louveau et al., 2018). Surprisingly, dampening meningeal immune cell drainage into the deep CLNs, either by meningeal lymphatic vessel ablation (Louveau et al., 2018) or surgical resection of the deep CLNs (Furtado et al., 2008; Louveau et al., 2018), improved EAE clinical symptoms. Conversely, ablation of the nasal lymphatics near the cribriform plate reduced T cell drainage into the superficial CLNs, but did not affect EAE development or progression (Louveau et al., 2018). These observations suggested that the nasal lymphatic drainage route might have a minor role in this disease model and that the deep CLNs seem to be a key site of encephalitogenic T cell generation (Louveau et al., 2018). Transcriptomic analysis of myelin oligodendrocyte glycoprotein (MOG)-specific T cells isolated from deep CLNs of mice with dysfunctional meningeal LVs reveled a lessened encephalitogenic profile, when compared to EAE mice with intact meningeal lymphatics, which might explain the observed differences in clinical scores (Louveau et al., 2018). However, it is important to emphasize that resection of the deep CLNs at an advanced symptomatic stage of EAE resulted in decreased number of myelin basic protein (MBP)-specific T cells infiltrating the CNS, but was not sufficient to improve the animals clinical score (Furtado et al., 2008). Likewise, neither meningeal lymphatic ablation nor resection of the deep CLNs were able to prevent the appearance of clinical symptoms in the EAE model (Furtado et al., 2008; Louveau et al., 2018), suggesting that other routes of immune cell drainage and reactivation might be involved in disease onset and progression.

The lymphangiogenic factor VEGF-C has also been associated to altered disease outcome in EAE models. Overexpression of VEGF-C (Park et al., 2013; Hsu et al., 2019) and its receptor VEGFR3 (Park et al., 2013) occurs in the spinal cord (Park et al., 2013) and brain (Hsu et al., 2019) of EAE mice, compared to controls. Inhibition of VEGFR3 signaling, by peripheral injection of the tyrosine kinase inhibitor MAZ51, delayed EAE onset and decreased disease severity, by reducing demyelination and infiltration of cluster of differentiation (CD) 4 T cells in the spinal cord (Hsu et al., 2019). It is still debatable whether this beneficial effect was achieved through the modulation of the meningeal lymphatics, the peripheral nasal lymphatics, or both systems (Louveau et al., 2018; Hsu et al., 2019). Nevertheless, similarly to what was observed upon deep CLN’s resection, treatment with MAZ51 at more advanced stages of EAE was not able to improve the clinical score (Hsu et al., 2019), which again implies that meningeal lymphatic drainage might play an important role in early stages of CNS autoimmune disease development, but a minor role in a later phase of disease.

### Brain Tumors

Primary brain tumors (like gliomas) rarely spread into peripheral tissues (Mondin et al., 2010; Lun et al., 2011), a phenomenon that might be explained, in part, by the lack of LVs in close proximity to tumor cells within the brain parenchyma (Mondin et al., 2010; Da Mesquita et al., 2018a). Additionally, it was recently reported that the subdural and striatal injection of glioma or melanoma cells in mice induced dorsal meningeal lymphatic remodeling (Hu et al., 2020) and that glioma-bearing mice show impaired CSF outflow through meningeal LVs (Ma et al., 2019c). The abnormal redirection of CSF flow towards the spinal cord and altered turnover of brain fluid around the tumor environment (Shibahara et al., 2019) might be a consequence of decreased meningeal lymphatic CSF outflow and impaired glymphatics, which can be favoring the accumulation of toxic proteins, cytokines and chemokines that enable tumor growth (Da Mesquita et al., 2018a). On the other hand, decreased CSF outflow may reduce the transport of tumor-derived antigens to the draining LNs, thus compromising the activation and proliferation of anti-tumor T cells (Alves de Lima et al., 2020). Interestingly, two recent studies have implicated the meningeal lymphatic system in the development of a protective immune response against tumor cells in the brain (Hu et al., 2020; Song et al., 2020). Enhancement of meningeal lymphatic drainage capacity by VEGF-C promoted the drainage of brain tumor cells into the deep CLNs and boosted anti-programmed cell death protein 1 (PD-1)/anti-cytotoxic T lymphocyte antigen 4 (CTLA-4)-mediated immunotherapy against intracranial tumors, without interfering with tumor angiogenesis or growth (Hu et al., 2020; Song et al., 2020). Importantly, the near-complete rejection of brain tumors by prophylactic VEGF-C overexpression was absent in mice with impaired lymphatic drainage into the deep CLNs (Song et al., 2020) or upon blockade of CCR7 signaling by CCL21, which directly implicates meningeal lymphatic drainage in these therapeutic approaches.

### Brain Injury and Hemorrhage

Modulation of meningeal lymphatic drainage was shown to affect the outcome of brain injuries, such as traumatic brain injury (TBI), subarachnoid hemorrhage and stroke. Induction of TBI in mice resulted in a substantial decrease in meningeal lymphatic drainage as early as two hours post-injury, which was only fully restored two months later (Bolte et al., 2020). In accordance, CSF flow was altered in TBI (Johanson et al., 2011), and intracranial pressure was markedly increased at two hours post-injury (Bolte et al., 2020). Photodynamic ablation of meningeal lymphatics prior to TBI exacerbated the neuroinflammatory response, including the overexpression of complement pathway genes, and aggravated the cognitive deficits triggered by the injury. On the other hand, improving meningeal lymphatic function in old mice by viral-mediated VEGF-C overexpression decreased neuroinflammation induced by TBI (Bolte et al., 2020).

Increased meningeal T cell numbers were observed in a mouse model of subarachnoid hemorrhage, which could be attributed to an impairment in the draining capacity of meningeal LVs (Pu et al., 2019). Surgical resection of the deep CLNs in a rat model of subarachnoid hemorrhage further reduced the regional cerebral blood flow and led to worsened intracranial pressure, brain edema, oxidative stress and motor impairment (Sun et al., 2006; Sun et al., 2011). Exacerbated neuroinflammation and impaired cognitive performance were also observed in a mouse model of subarachnoid hemorrhage upon photodynamic ablation of meningeal lymphatics or injection with MAZ51 (Chen J. et al., 2020). Likewise, ligation of the collecting lymphatics draining into the deep CLNs resulted in worsened outcome in a model of subdural hematomas (Liu X. et al., 2020).

Increased VEGF-C levels were observed in the CSF and brain parenchyma of two different animal models of stroke (Gu et al., 2001; Esposito et al., 2019), pointing to a potentially augmented VEGFR3 signaling in lymphatic vasculature (Esposito et al., 2019). However, different stroke models have resulted in somewhat contradictory observations in terms of meningeal lymphatic remodeling and role. Meningeal lymphangiogenesis was observed in a photothrombosis-induced focal stroke model, but not in a stroke model induced by transient middle cerebral artery occlusion (MCAO) (Yanev et al., 2020). While it was initially reported that surgical blockade of lymphatic drainage into the CLNs led to increased infarction volume in a model of MCAO (Si et al., 2006), a more recent report has shown a conflicting reduction in infarction volume upon MCAO and resection of the superficial CLNs or MAZ51 treatment (Esposito et al., 2019). In addition, defective lymphatic function by *Vegfr3* haplodeficiency resulted in larger infarct volumes after MCAO, but not after photothrombosis-induced stroke (Yanev et al., 2020). Interestingly, an elegant study using different zebrafish models of brain thrombosis has described a transient formation and extension of lumenized LVs from the meninges into the injured brain parenchyma (Chen et al., 2019). These lymphatics were involved in fluid drainage, reduction of cerebral edema, and regressed shortly after cerebrovascular regeneration so that the brain parenchyma could return to its homeostatic lymphatic-free status (Chen et al., 2019). It remains to be investigated if the same mechanisms of transient meningeal lymphangiogenesis occur, or can be induced, in the mammalian injured CNS and whether this process supports the resolution of neuroinflammation and restoration of neuro-glio-vascular function upon injury, hemorrhage or stroke.

### Hepatic Encephalopathy

Hepatic encephalopathy is a neurological complication that results from cirrhosis and causes cognitive, psychiatric and motor impairment (Weissenborn, 2019). Aberrant neuroinflammation is a common feature observed in animal models of hepatic encephalopathy (Liu R. et al., 2020; Izquierdo-Altarejos et al., 2020). Rats subjected to bile duct ligation, a hepatic encephalopathy disease model, presented increase meningeal lymphangiogenesis that was further stimulated by increasing VEGF-C levels in the CSF (Hsu et al., 2020). Interestingly, boosting meningeal lymphangiogenesis led to a down-regulation of proinflammatory genes, attenuated microglial activation in the brain cortex, and improved motor function (Hsu et al., 2020). Collectively, these results suggest that a faster resolution of brain inflammation through enhanced meningeal lymphatic drainage could improve hepatic encephalopathy.

## Meningeal Lymphatics in Brain Health and Disease: Future Challenges

The seminal (re)discovery of the meningeal lymphatic vasculature (Aspelund et al., 2015; Louveau et al., 2015) opened the door for an array of novel experimental data that have contributed to advance our knowledge about the mechanisms governing CNS lymphatic drainage. However, every significant step towards a more in-depth understanding of the meningeal lymphatic system in health and disease (Figure 2), has been accompanied by fresh, interesting and puzzling questions. We will now summarize some open questions and hypothesis, which have been fueled by recent experimental evidence about the meningeal lymphatic system, brain aging and neurological disorders.

Aging has a profound impact on the meningeal lymphatics (Da Mesquita et al., 2018b; Ahn et al., 2019). However, the effect of aging is not exclusive to the CNS or its lymphatic vasculature, but systemic, affecting the blood composition and many peripheral tissues (Villeda et al., 2011; Chen M. B. et al., 2020; Schaum et al., 2020). The meningeal dural lymphatics are located right next to the fenestrated venous sinuses, which are quite permissive to the exchange of both molecules and cells between the blood and the stroma of the meninges (Balin et al., 1986; Alves de Lima et al., 2020). This means that an aged systemic milieu (Villeda et al., 2011; Chen M. B. et al., 2020; Schaum et al., 2020) characterized by altered levels of plasma solutes and proteins, as well as circulating immune cells, alongside a hypothetical loss of blood-meningeal integrity with aging (Rustenhoven et al., 2021) might take a significant toll on the meningeal lymphatic system.

While the cellular and molecular culprits of age-related meningeal lymphatic dysfunction remain unclear, the data gathered so far implicates the VEGF-C–VEGFR3 signaling pathway as a mechanism that can be therapeutically exploited to rescue meningeal lymphatic drainage in the aged CNS (Da Mesquita et al., 2018b). However, we know very little about the cellular sources of meningeal VEGF-C, or how aging impacts on VEGF-C production and maturation in the meningeal compartment. In fact, the same premises apply for other growth factors, cytokines or signaling pathways that have the ability to shape meningeal LEC function, namely VEGF-D (Achen et al., 1998), TNF and interleukin (IL)-1β (Schwager and Detmar, 2019), and altered growth factor signaling by WNT, BMP2 or through neuropilin 2 (Yuan et al., 2002; Dunworth et al., 2014; Cha et al., 2018). Moreover, it will be worth testing whether the morphological and functional changes in meningeal lymphatics with aging result from a dysregulated expression of the transcription factors FOXC2 and GATA2 due to changes in shear stress or extracellular matrix composition at the dural layer (Sabine et al., 2015; Sweet et al., 2015; Da Mesquita et al., 2018b; Rustenhoven et al., 2021). The loss of initial lymphatics in the meninges might also be explained by a reduced renewal capacity. Future studies should focus on the regenerative capacity of the meningeal lymphatic system in adulthood and aging, with a special emphasis on the cellular progenitors that can give rise to LECs [extensively reviewed in (Gutierrez-Miranda and Yaniv, 2020)] in the meningeal compartment. A loss of particular types of meningeal LEC’s progenitors with aging may account for the change in meningeal lymphatic coverage and morphology, either in “normal” aging or in certain diseases of the CNS.

As previously highlighted in this review, defects in meningeal lymphatic output result in dampened glymphatic function (Da Mesquita et al., 2018b) and, consequently, in reduced excretion of brain molecular waste products into the subarachnoid CSF. This phenomenon could explain, at least in part, the accumulation of toxic Aβ, Tau or α-synuclein species in the brains of different models of neurodegenerative disorders (Da Mesquita et al., 2018b; Patel et al., 2019; Wang et al., 2019; Zou et al., 2019). However, the exact mechanisms governing the interactions between the meningeal lymphatics and the cellular components of the glymphatic perivascular route (astrocytes, perivascular macrophages, pericytes, SMCs, or blood endothelial cells, just to name a few) are still unknown [reviewed in (Da Mesquita et al., 2018a)]. The anatomical segregation between the meningeal lymphatic vasculature and the brain parenchymal cells (supposedly separated by a restricting blood-meningeal barrier), makes it even more challenging to pinpoint the molecular mediators underlying the crosstalk between the meningeal lymphatic system, brain phagocytes and the neurovascular unit (that shares cellular types with the glymphatic system), three important players in brain cleansing (Da Mesquita et al., 2018a; Sweeney et al., 2018a; Shi and Holtzman, 2018; Alves de Lima et al., 2020). Deciphering which specific brain cell types are more susceptible to reduced meningeal lymphatic function (and when it happens) might prove to be a herculean task, specially taking into consideration the wide effects of meningeal lymphatic dysfunction on fluid/macromolecule perivascular circulation throughout the whole brain (Da Mesquita et al., 2018b). One possible starting point will be to focus on processes that are closely involved in regulating the levels of Aβ and Tau aggregates in the brain, including the molecular transport mechanisms across the BBB (Sweeney et al., 2018b), or even glial and neuronal activation. Hypothetically, a decrease in meningeal lymphatic function could indirectly impact on the circadian levels of Aβ and Tau in the brain’s ISF and CSF by shaping neuronal excitation and/or inhibition (Hong et al., 2011; Yamada et al., 2011). Alternatively, the meningeal lymphatics could be involved in the regulation of the sleep-awake cycle, which has a profound influence on brain processes that regulate the levels of extracellular toxic Aβ and Tau species in fluids and can affect their accumulation and aggregation in the brain (Iliff et al., 2012; Roh et al., 2012; Xie et al., 2013; Holth et al., 2019).

The neuroimmune system is a major player in brain aging and certain neurological disorders. However, it is still debatable whether meningeal lymphatic dysfunction is contributing to the changes observed in CNS immunity with aging. Reduced immune cell egress through lymphatics can be contributing to the higher frequency of T cells and altered macrophage and DC phenotypes observed in the aged meninges and brain (Mrdjen et al., 2018). Alongside the T cell pool expansion, there is an increased T cell clonality in the aged CNS, namely in the choroid plexus and at the vicinity of the brain ventricles and neurogenic niches, that was associated with deleterious changes in neuroinflammation and neurogenesis (Baruch et al., 2013; Dulken et al., 2019). Interestingly, changes in the response by T cells were also observed in human neurodegenerative diseases. Increased reactivity to α-synuclein peptides by peripheral helper and cytotoxic T cells from PD patients was detected immediately after the diagnosis of motor symptoms (Sulzer et al., 2017; Lindestam Arlehamn et al., 2020). Along the same line, clonally expanded CD4 and CD8 T cells specific to two Epstein–Barr virus antigens were found in the CSF of AD patients (Gate et al., 2020). The next step will be to perform functional experiments in order to better understand whether these changes in T cell clonality impact disease severity or outcome. Importantly, future studies should also investigate what drives the phenomena of increased T cell clonality and reactivity in neurological diseases: we postulate that altered lymphatic drainage of CNS-derived antigens (Aβ, Tau, α-synuclein or persistent virus) and/or reduced immune cell egress (of T cells or antigen presenting cells) through meningeal lymphatics into the CLNs with aging (Figure 2), might contribute to the above-mentioned changes in CNS adaptive immunity. It is also possible that the aging-induced loss of initial LVs at the meningeal dural layer and at the CNS-draining CLNs results in decreased levels of certain chemokines, such as CCL19, CCL21 and CSF1, that are crucial for proper immune cell egress and surveillance of the CNS (Louveau et al., 2016; Mondor et al., 2019). Accordingly, improving meningeal lymphatic function in the aged or diseased CNS might represent a promising therapeutic method to revert an atypical immune response and resolve deleterious neuroinflammation. Treating aged mice with VEGF-C resulted in better cognitive performance as a direct consequence of improved lymphatic drainage into the deep CLNs (Da Mesquita et al., 2018b). It will be informative to experimentally assess whether the beneficial effects of VEGF-C were achieved by improved drainage of CSF solutes alone, by a fine-tuning of meningeal/brain immunity, or if both components were equally improved in old mice and contributed to better brain function.

Recent studies have linked defects in meningeal lymphatic drainage to exacerbated gliosis and overexpression of innate immune genes in models of TBI, brain α-synucleinopathy and hepatic encephalopathy (Zou et al., 2019; Bolte et al., 2020; Hsu et al., 2020). The next step will be to decipher the exact mechanisms through which meningeal lymphatics can shape the brain innate immune response. Brain resident innate myeloid cells, namely microglia and perivascular macrophages, play very important roles in response to traumatic insults and in disorders of different etiologies-for instance in EAE, AD and brain tumor models-showing in some cases disease-specific activation profiles (Keren-Shaul et al., 2017; Muller et al., 2017; Ajami et al., 2018; Kang et al., 2018). The fact that the meningeal lymphatic system has been closely implicated in the pathophysiology of similar models of disease (Da Mesquita et al., 2018b; Louveau et al., 2018; Hu et al., 2020; Song et al., 2020), emphasizes the need of exploring the possible connections (direct or indirect) between altered meningeal lymphatic function and abnormal brain innate myeloid cell activation.

All of the above-mentioned premises and hypothesis around meningeal lymphatics remain to be tested and might have major implications for the basic understanding of the pathophysiology of CNS autoimmune diseases and tumors, AD, PD and other neurological diseases. In sum, we are still a long way from knowing all the mechanisms governing meningeal lymphatic maturation, morphology and drainage, and if meningeal lymphatic function can be harnessed to achieve effective therapeutic outcomes in the aged and/or unhealthy CNS.
